# Debate: Standing up for science – how to combat misinformation in child mental health? The imperative to understand and counter mental health misinformation on social media

**DOI:** 10.1111/camh.70081

**Published:** 2026-03-11

**Authors:** Lorenzo Lorenzo‐Luaces, Isabella Starvaggi

**Affiliations:** ^1^ Indiana University‐Bloomington Bloomington IN USA

**Keywords:** Social media, misinformation

## Abstract

Children and adolescents spend a large amount of time on social media, where they often seek health information. Growing research suggests misinformation is common across various domains of health, such as infectious disease and men's sexual health. Burgeoning evidence suggests that misinformation about mental health may be common as well, especially with regard to trauma and neurodevelopmental diagnoses. There is a need to understand the real‐world context that facilitates the spread of misinformation as well as what features of the online environment promote misinformation. Researchers and clinicians working with children and adolescents must consider the role of misinformation in their work. All mental health professionals need to disseminate accurate, helpful, and engaging information about mental health and mental health treatments.

Ambiguous or misleading information, also known as misinformation, about mental health on social media is an underrecognized and under‐researched topic (Starvaggi, Dierckman, & Lorenzo‐Luaces, 2024). Rates of internet access vary worldwide. However, most individuals with internet access use social media, and youth tend to be more active on social media than adults. For example, 46% of US adolescents use at least one social media platform ‘almost constantly’ (Faverio & Sidoti, 2024). Misinformation about politics and physical health on social media and its societal impacts is well‐documented, most notably regarding vaccines (cf. Starvaggi et al., 2024).

Misinformation about mental health on social media may have serious societal implications (Starvaggi et al., 2024), particularly for how youth conceptualize mental health and its treatment. For example, a 74‐second TikTok video claims one can ‘release stored trauma’ in the body by doing hip thrusting exercises. The video has been viewed nearly 300,000 times in the past 3 years, under a ‘somatic therapy’ hashtag. ‘Somatic therapy’ refers to a set of putatively therapeutic approaches claiming to address post‐traumatic stress disorder (PTSD) symptoms by focusing on how the body physically ‘stores’ trauma. Other somatic therapy videos directly state that treatment for PTSD and trauma must include a ‘somatic therapy’ component if it is to be effective. There have been at least two randomized controlled trials attempting to study the efficacy of somatic therapies for PTSD (Kuhfuß, Maldei, Hetmanek, & Baumann, 2021). However, to date, somatic therapy has no strong evidence of efficacy for trauma or stressor‐related disorders. Thus, online content making unqualified claims about the efficacy of ‘somatic’ techniques for traumatic stress is misleading and potentially harmful.

## The scope of the problem

Adolescents use the internet, including social media, as a source of health information (Park & Kwon, 2018). The quality of health information on social media is generally poor, but its reach is enormous. Research documenting misinformation about mental health is in its early stages, but high rates of misinformation have been documented, especially in relation to popular topics like trauma and neurodevelopmental diagnoses like autism (Starvaggi et al., 2024). Content can also be potentially harmful through misleading messaging. For example, popular TikTok videos about ADHD and autism have content that is mostly inaccurate, lacks scientific evidence, or is full of overgeneralizations, particularly regarding signs and symptoms of neurodevelopmental disorders (e.g., Aaragon‐Guevara et al., 2025; c.f. Starvaggi et al., 2024).

Once misinformation starts spreading, it spreads quickly. This is because viral online content views follow a ‘power law’ distribution wherein a small number of posts are seen by many users (e.g., ‘viral videos’). In one study, Twitter/X posts about monkeypox were replete with misinformation, like the claim that monkeypox *exclusively* occurs in men who have sex with men (Edinger et al., 2023). This inaccurate claim was pernicious with harmful and stigmatizing implications. When minors were diagnosed with monkeypox, online communities argued they were the victims of sexual abuse, promoting harmful stereotypes about men who have sex with men. The World Health Organization (WHO) attempted to counter misinformation by disseminating accurate information. Even though it only took them 2 weeks to do so, by that time, it was nearly impossible to ‘catch up’ to the viral spread of misinformation. Organizations like the WHO may stand little chance of developing educational content as popular and sensational as misinformation, which may achieve virality by stoking outrage and negative emotionality (McIntyre, 2021), underscoring the need for *rapid* responses.

A startling example of harmful health misinformation is a social movement that discourages and pathologizes masturbation in young men with the hashtag ‘#noFap’. Posts claim that ‘fapping’, slang for ‘masturbating’, leads to mental health problems like depression or pornography addiction, and physical problems like muscular weakness and erectile dysfunction (Prause & Binnie, 2024). Forums contain disturbing suggestions of self‐harm as solutions to ‘relapses’ including chemical castration and even suicide. Many #noFap users also engage with ‘incel’, or ‘involuntarily celibate’, communities, pointing to broader societal harms. Most #noFap forum participants see misogynist content, and approximately half report bullying and anti‐LGBT content, underscoring this community's harms to youth physical and psychological wellbeing.

## Understanding what and why

Some misinformation is spread maliciously with a deliberate intent to mislead (‘disinformation’), as documented in politics. Intentions to mislead may be present in mental health content, although the determination that social media content is misinformed can be complex. For example, posts often contain paid content like advertisements. The ‘somatic therapy’ video we referenced earlier contains a link to a paid 30‐day program with somatic exercises to ‘cure’ trauma. Nonetheless, not all misleading content is malicious. Content should be interpreted with nuanced consideration of the social‐cultural context in which it is produced, including the users' experiences with the limitations of mental health services (Starvaggi et al., 2024). For example, it is possible that the popularity of social media content about autism self‐diagnosis is related to the inaccessibility of autism assessments and the historical underdiagnosis of individuals from marginalized groups. Social media content also often contains overgeneralizations or anecdotal accounts that are not completely false, which may have the potential to be misleading but also informative. For example, one study found that reports of rare negative side effects of antidepressants are common on Instagram (c.f. Starvaggi et al., 2024). Similarly, our work has suggested that negative content about cognitive behavioral therapies (CBTs) often includes anecdotes of negative first‐hand experiences with CBT (Lorenzo‐Luaces, Dierckman, & Adams, 2023). On the one hand, these personal stories may inadvertently lead to potentially harmful treatment decisions (e.g., not pursuing CBT when one may benefit from it). On the other hand, the popularity of this content may reflect the field's longstanding ignorance of potential harms of psychological interventions. Therefore, to understand whether and what mental health content on social media is misleading, it may be important to consider its broader social and cultural context.

Studying how misinformation spreads is difficult because of the ‘black box’ nature of the algorithms that drive social media content, and disincentives to divulge their features (Bak‐Coleman, O'Connor, Bergstrom, & West, 2025). However, user engagement (e.g., views) is known to influence algorithms (‘engagement begets engagement’). Therefore, understanding why certain topics are appealing to users may inform efforts to combat misinformation. Content like ‘somatic’ therapy videos promises quick and easy solutions (e.g., hip exercises) to longstanding problems (e.g., PTSD). The wide appeal of such posts could be driven by high unmet mental health needs and prohibitive barriers to accessing legitimate treatment, both of which are well‐documented. It might be beneficial to design counter‐misinformation efforts that offer alternative, accessible avenues for help, rather than information alone.

Experiments have revealed effective methods to combat misinformation (McIntyre, 2021). At least two appear generally, and roughly equally, effective. One strategy is to provide accurate content (e.g., ‘vaccines do not cause autism’) and the other is to teach individuals about misinformation techniques like cherry‐picking data. Preventive strategies can also help youth be inoculated against misinformation, for example, by promoting skepticism of content made by non‐experts. Clinicians should be armed with these tools to prevent and combat misinformation. However, given the extent to which youth engage with social media, it may be prudent for clinicians to assume that youth have encountered mental health content on social media and, proactively and by default, assess youths' understanding of their illness and its treatments. In other words, rather than waiting to hear misinformation in the therapy room, competency conducting psychotherapy with youth may require understanding that their digital environments are replete with misinformation. It also appears imperative that clinicians, scientists, and individuals with relevant mental health expertise lead contemporary conversations on child and adolescent mental health by disseminating accurate, helpful mental health information in the social media environment.

## Conclusion

Youth increasingly seek mental health information on social media. Growing evidence suggests they are exposed to abundant misinformation. Even when misinformation is a small percentage of all content on a topic (e.g., vaccines), that small amount may lead to real‐world harm. There is an obvious need to leverage social media to disseminate accurate, and helpful, information about child and adolescent mental health. We urge practitioners and researchers to recognize the urgency of this public health problem and leverage their expertise to combat it.

## Funding information

This research was partially supported by a National Institutes of Health grant R01‐MH‐135502 (PIs: Lorenzo‐Luaces & J. Bollen). The sponsor had no role in the drafting of the current manuscript.

## Conflict of interest statement

The authors declare no conflicts of interest.

## Ethics statement

No ethics approval was required for this debate article.

## Data Availability

Data sharing is not applicable to this article, as no datasets were generated or analyzed for this debate article.
